# Family functioning and stroke: Family members’ perspectives

**DOI:** 10.4102/ajod.v10i0.801

**Published:** 2021-10-25

**Authors:** Sibulelo Gawulayo, Charlene J. Erasmus, Anthea J. Rhoda

**Affiliations:** 1 Department of Social Work, Faculty of Community and Health Sciences, University of the Western Cape, Cape Town, South Africa; 2 Centre for Interdisciplinary Studies on Children, Families and Society, Faculty of Community and Health Sciences, University of the Western Cape, Cape Town, South Africa; 3 Department of Physiotherapy, Faculty of Community and Health Sciences, University of the Western Cape, Cape Town, South Africa

**Keywords:** stroke, stroke survivor, stroke impairments, activities of daily living (ADL), family members, family functioning and dimensions

## Abstract

**Background:**

Stroke survivors often experience permanent or temporal physical and psychological stroke impairments. As a result, stroke survivors are often discharged to recover in their home environments and are cared for mostly by family members. Additionally, caregiving roles are often assumed without any formal training or preparation whatsoever. This can transform the family’s functional patterns due to adjustments that are made to accommodate the caregiving needs.

**Objectives:**

To explore the experiences and influence of stroke on families and on family functioning.

**Method:**

Explorative descriptive qualitative research design through the use of in-depth interviews were employed as the means of data collection. The sample size was eight (8) family members and was guided by the saturation point. Data was thematically analysed.

**Results:**

Four themes emerged from the analysis: 1) reduced interactions with family members due to communication barriers, 2) the influence of stroke on family relationships, 3) emotional engagement in caring for a family member with a stroke and 4) financial implications of stroke on family functioning. This study found that stroke can influence the family functioning negatively as family members may be forced to change their functional patterns. However, some family members reported positive experiences, they developed a supportive structure to accommodate the new life of the stroke survivor.

**Conclusion:**

Using the McMaster’s model of family functioning, this study found that stroke is a threat to the six dimensions of family functioning: 1) problem-solving, 2) communication, 3) roles, 4) affective responsiveness, 5) affective involvement, and 6) behaviour control.

## Introduction

Stroke is a public health concern with a devastating impact on the individual’s health and well-being, especially in developing countries such as South Africa (Kumar, Kaur & Reddemma 2015; Rhoda 2014). The sudden occurrence of stroke is shocking and can be life-changing, leaving the stroke survivor and their family unprepared for dealing with its aftermath (Lutz & Yound 2010). Worldwide, stroke is responsible for approximately 5.7 million deaths annually (Kim & Johnston 2013). In South Africa, stroke is responsible for 25 000 deaths annually and more than 95 000 survivors live with disabilities caused by stroke (Taylor & Ntusi 2019). The most common neurological dysfunctions caused by stroke include mobility, speech problems and the inability to perform activities of daily living (ADL) (Ramos-Lima et al. 2018), such as home management, self-care, work and social roles (Ahn & Hwang 2018). As a result of decreased physical functioning, stroke survivors may find it difficult to actively participate or engage in social activities (Whitiana, Vitriana & Cahyani 2017). This may lead to social isolation and contribute to depression and feelings of helplessness (Brookfield & Mead 2016). As a result, most stroke survivors are often assisted by their family members to attain ADL. Furthermore, the overburdened South African public healthcare system struggles to provide adequate support for stroke survivors, thereby passing the caregiving responsibilities to their families (Maredza & Lumbwe 2016). Thus, maintaining supportive ties with family members, relatives and close friends has been found to exert a positive influence on the life of the stroke survivor (Kumar, Kaur & Reddemma 2016; Rhoda 2014). However, because of the unexpected transitions from providing care and support to the stroke survivor (Jiang et al. 2014), the family functioning of such families is often threatened (Gillespie & Campbell 2011). Therefore, this article discusses the family functioning following the stroke of a family member.

Upon discharge, stroke survivors are often cared for by their families who ensure that the home environment is conducive enough to support the recovery process (Whitiana et al. 2017). When first receiving the stroke diagnosis, the family is often confused and worried about the life of their family member. An acute state of distress is typically followed by feelings of shock, disbelief, fear and anxiety because of uncertainties about the future (Lutz et al. 2011). Although this is the case, helping the stroke survivor to achieve ADL becomes a priority to families of stroke survivors. Activities of daily living include the everyday activities such as bathing, eating, dressing, toileting, bladder and bowel control, mobility, transfers and the ability to climb stairs (Rhoda, Mpofu & De Weerdt 2011:16) these activities are often identified as self-care. An additional noteworthy point is that the process of caring for the stroke survivor is very demanding and can take a physical and psychological toll on the caregiver, leading to burnout (Gillespie & Campbell 2011; Kumar et al. 2016). Caring for the person with stroke also has a detrimental impact on the well-being and overall quality of life of family members (Guo & Liu 2015). This is further supported by Gillespie and Campbell (2011) who found that the caregiving and all due considerations post-stroke have a negative effect on family functioning.

Family functioning – a central concept in this study – refers to the family’s day-to-day patterns that are practiced within the family context to enable favourable conditions for family members to thrive (Mousavi et al. 2015) and represents the family’s capacity to ensure that the basic and essential needs of its members are met (Gunaratne 2008). Dai and Wang (2015) identified that the dimensions that inform family functioning are tasks and roles, communication patterns, emotional expression, involvement, behaviour and values and rules. Chronic diseases have a considerable negative effect on the lives of family members as their day-to-day functioning may change or be disrupted (Golics et al. 2013). In this regard, families struggle to maintain a stable environment for the stroke survivor and other family members while providing caregiving (Lutz et al. 2011). Similarly, Gillespie and Campbell (2011) found that post-discharge from the healthcare facility, families explore various measures to accommodate the stroke survivor’s needs.

In seeking to determine appropriate measures to accommodate the new life of the stroke survivor, areas of family functioning, including financial well-being, may be strained (Ramos-Lima et al. 2018). Botha, Wouters and Booysen (2018) found that the financial well-being of the family is an important aspect that measures the family’s ability to care for the material needs of its members. King et al. (2010) opined that sustaining financial stability post-stroke is a challenge as family members struggle to find a balance between work and caregiving. They are often absent from work to care for the stroke survivor. For the most part, Kumar et al. (2016:58) reported that ‘caregiving tasks typically require time and energy for months and even years together’. Consequently, the latter may compromise the family’s ability to cover caregiving costs that include, amongst others, the recommended diet and transportation costs for outpatient therapy services (King et al. 2010; Ski et al. 2015).

Another dimension of family functioning impacted by the incident of stroke is family relationships. Children and spouses often experience altered relationships with their parents and life partners. Parents who experienced a stroke may be limited to assume their parental responsibilities post-stroke (Harris & Prvu Bettger 2018). In addition, stroke has a significant effect on marital or partner relationships. A study conducted by Kitzmüller, Asplund and Häggström (2012) reported that:

[*B*]oth stroke survivors and spouses experience role changes, an altered perception of self and a loss of social activities, and these changes influence both personal identity and the dynamics of spousal relationships. (p. 1)

It is therefore apparent that stroke alters family relationships as the family progresses to establish a new lifestyle. In addition, stroke survivors often experience communication deficits that result in speech problems, such as aphasia, dysarthria or apraxia. Regarding stroke-related communication challenges, Wray and Clarke (2017:2) stated:

Up to one-third of survivors will experience communication difficulties post-stroke including aphasia, dysarthria or apraxia of speech 12–15 resulting in difficulties with language comprehension, speech production and difficulties with reading and writing.

Families of stroke survivors may thus find it difficult to maintain good communication, although not necessarily because of the disease itself, but because of the psychological impact and coping strategies, which affects how family members react towards each other (Guo & Liu 2015).

In light of the given discussion, family functioning is an important area that must be safeguarded post-stroke as the family plays a pivotal role in the stroke survivor’s recovery (Creasy et al. 2015). However, a relatively unexplored area that appears to have received little attention is the impact of stroke on the dimensions of family functioning. Information on how families and family members respond to the incident of stroke is important and must be explored to gain insight into their perceptions and experiences (Bryer et al. 2010) to optimise family functioning, make improvements where necessary and prevent overburden of family caregivers. Research, both local and international (Cameron et al. 2014; Elloker & Rhoda 2018; Hassan, Visagie & Mji 2011; Kubina et al. 2013; Ski et al. 2015), has focused more on the experiences of caregivers and the relationship between social support and participation post-stroke. Studies on stroke (Khondowe, Rhoda & Mpofu 2007; Simeone et al. 2015; Wagachchige-Muthucumarana, Samarasinghe & Elgán 2018) have explored the experiences of caregivers, with few focusing on the impact of stroke on family functioning.

To our knowledge, information that documents the family’s experience of stroke and the influence of stroke on family functioning is limited. Elloker and Rhoda (2018) explored factors that contribute to social support and participation post-stroke. On the other hand, extensive research focused on the caregiver’s experiences of caring for individuals who experienced a stroke (Hassan et al. 2011; Khondowe et al. 2007; Kumar et al. 2016). This article therefore hopes to shed light on the influence of stroke on family functioning within the South African context, as this is often an overlooked research area.

### Conceptual framework

The McMaster’s model of family functioning and the family systems theory is used to conceptualise the functioning of families with a stroke survivor within the six dimensions, namely (1) problem-solving, (2) communication, (3) roles, (4) affective responsiveness, (5) affective involvement and (6) behaviour control (Miller et al. 2000; Mousavi et al. 2015). These dimensions determine a conducive environment where family members develop relationships and establish emotional connections in order to withstand life’s shortcomings. Ensuring a conducive environment during stroke is a challenging task that disrupts normal family functional patterns (Przewoźnik, Rajtar-Zembaty & Starowicz-Filip 2015). In a study that explored the influence of stroke on families, Gillespie and Campbell (2011) and Guo and Liu (2015) found that establishing a secure caring environment for the stroke survivor is a tiresome process that requires families to adjust their normal functioning, which impacts negatively on the dimensions of family functioning, such as family communication, individual roles, involvement and family relationships.

McMaster’s model of family functioning gives an overview of family functioning based on the aforementioned dimensions. In addition, the model gives a systematic overview of the family functioning. The proposed dimensions of the family functioning guided the development of the interviewing guide and the presentation of findings. An interviewing guide is attached in this article, see Appendix 1. There are no studies that have been conducted that look into the family functioning of stroke survivors using the McMaster’s model of family functioning. However, the model can be used to explore the functioning of families following a tragic event (Boterhoven de Haan et al. 2015; Fogarty 2009). Both studies found that family functioning is an important part of our lives with a significant contribution to our day-to-day functioning. This model is therefore relevant in this study.

## Methodology

### Research design

An explorative and descriptive research design was used to explore the influence of stroke on family functioning. Such a design is mainly used to gather new information and to create meaning out of participants’ lived experiences (Blanche et al. 2006). Face-to-face in-depth individual interviews were conducted to collect data guided by a predetermined interview guide. The latter is a pre-planned set of questions, designed by authors of this study to collect data and guide the process of data collection (Akaranga & Makau 2016). All participants were afforded full opportunity to express their views regarding the influence of stroke on their family’s functionality.

### Research setting

This study was conducted in a peri-urban community in Cape Town, South Africa. Despite the fall of apartheid in 1994, the conditions in many South African communities remain unaltered because of slow economic development. Rapid intervention is therefore needed to improve these dire conditions (Charman 2017). The population of this study consisted of African people residing in the under study setting. Participants were accessed from a community-based organisation that provides psychosocial support to stroke survivors and their families to cope with stroke.

### Participants

Eligible participants included family members of stroke survivors who assisted them with ADLs. The inclusion criteria for participation required that participants had to (1) be a family member of a stroke survivor, (2) be residing in the peri-urban community under study and (3) have been providing care for at least 6 months, such as assisting them to assume ADL. Research found that the first 6 months of caring for a stroke survivor is considered the most crucial and challenging period, as the focus is on establishing the best possible care (Torregosa, Sada & Perez 2018) and adjusting family routines and processes to accommodate the stroke survivor. The sample size of this study consisted of eight family members (which included parents, children, siblings, and spouses) of a stroke survivor.

#### Participants’ characteristics

Eight participants (P) (five female and three male), with a mean age of 48 years, took part in the study. The highest level of education was a diploma and lowest was Grade 7 (entry level to high school in South Africa). The participants were closely related to the stroke survivor and included children, siblings, spouses and parents. Two of the eight participants were employed on flexible contracts and six were unable to work because they had to care for the stroke survivors. Lastly, participants in this study were predominantly African people. They represent peri-urban population in the Western Cape. Appendix 2 is a form that was used to collect the demographic information of participants.

### Data collection

An information session was conducted to explain the purpose of the study and to initiate contact with suitable participants. Data were collected through face-to-face individual interviews. Participants indicated that they would like to be interviewed in the comfort of their own homes and arrangements were made accordingly. All the interviews were audio-recorded, and field notes were taken after obtaining permission from the participants. Interviews were conducted in isiXhosa, participant’s home language and transcribed verbatim by an independent transcriber.

### Data analysis

An inductive bottom-up approach of data analysis as proposed by Creswell and Plano Clark (2007) was followed as it allows the researcher to group and organise the data and make sense of it by creating meaning. The presentation of themes followed Tesch’s eight steps of analysing and reporting qualitative findings (Theron 2015). The interviews were transcribed verbatim and checked to verify the accuracy of each participant’s exact words. Verifying and checking transcripts promotes rigour and accurate reporting of the research findings (Birt et al. 2016). Following the suggestion of Theron (2015), the researcher read and re-read the transcripts in order to formulate a general sense of the participants’ experiences. The latter led to the identification and grouping of similar experiences as categories and codes. The initial analysis identified five main major categories, underpinned by 11 codes. This includes, category (1) family’s reaction to stroke with no codes, category (2) caregiver’s experiences with four codes, category (3) experiences of living with a stroke survivor with four codes, category (4) influence of stroke on the family consisting of three codes and lastly, category (5) the needs of families of stroke survivors with no codes. These were further refined and grouped as themes and sub-themes. Four themes concluded the findings of this study.

### Ethical considerations

This study was ethically approved by Senate Higher Degrees Committee (reference number: HS17/10/20) of the University of the Western Cape. Furthermore, access to participants was granted by Helderberg Stroke Support Group (HSSG). Information sessions were conducted by the researchers to orientate the participants to the study, explain what would be expected of them and to assure them that privacy and confidentiality would be upheld. Participation in the study was voluntary, without any personal gain or remuneration. Participants were also free to withdraw from participation at any stage without fear of being penalised. In addition, participants were afforded an opportunity to clarify how they would like to be addressed during contact time with researchers in respect of their cultural terms. There was no potential harm detected in the planning and execution phase of this study. External counselling referral was secured prior to the execution of this study. Participants’ involvement was completely confidential and pseudonyms were used to protect their identities and no personal information was disclosed. All interview data were stored on a password-protected computer with the password known only to the researcher. Data will be disposed of in a suitable manner after 5 years as prescribed by the institution.

### Trustworthiness

Qualitative research needs to ensure rigour and trustworthiness in the reporting of the study’s findings (Hadi & Closs 2015). Researchers used the member checking to support the confirmability of the study’s findings. A summary of the field notes taken during data collection was shared with the participants to confirm if accurate representation of the experiences was captured. In addition, audio-recorded interviews were transcribed by an independent transcriber. Furthermore, the researchers compared the filed notes and transcripts from the independent transcriber to verify data accuracy. Verbatim quotes of participants’ accounts of their experiences of the influence of stroke on their family’s functioning are incorporated to ensure credibility. These processes were conducted to verify the accurate representation of the participants’ experiences. However, the findings are contextual and may not necessarily be applicable to other contexts.

## Findings

Four themes emerged from the analysis. Themes are as follow: (1) reduced interactions with family members because of communication barriers, (2) the influence of stroke on family relationships, (3) emotional engagement in caring for a family member with a stroke and (4) financial implications of stroke on family functioning. Each theme is discussed and supported with a verbatim quotation of the participant’s experiences. A discussion of the findings in the light of literature is presented.

### Reduced interactions with family members because of communication barriers

A total of 80% of the participants reported that they struggle to communicate with the family member who experienced a stroke because of speech problems. As a result, some participants reported that because of communication barriers, stroke survivors would often withdraw from interacting with other family members:

‘My mother used to be a talkative person. However, after the stroke she tends to withdraw herself from interacting with people.’ (P1, female, 42 years old)

‘There is not much that we do now because she likes her space and does not engage much with me. This worries me sometimes because there are times when I miss talking to her or sharing something together.’ (P4, male, 48 years old)

When they cannot attain the goal of communication, some stroke survivors would become easily frustrated, especially when they are not understood. A common concern shared by the family members was the imbalance of emotions expressed by stroke survivors, such as being agitated and easily irritated.

‘Our big challenge with him is communication. He has a very short temper. I try to take my grandchildren away during weekends to visit my sister so they may not interact with him.’ (P2, female, 52 years old)

‘My son does not talk much; however, I can see that he is bothered about his mother’s condition.’ (P4, male, 48 years old)

### The influence of stroke on family relationships

This study found that family relationships transform post-stroke incident. Participants particularly described how stroke influenced the quality time and also affected relationships in different levels, these include relationships with children, spouses and with extended family members. Family quality time refers to the time family members spend together during mealtimes, watching a television programme, playing games as a family or engaging in any interactive activities. Participants mentioned that post-stroke, families no longer spent time together or enjoyed quality time as they did prior to the stroke.

‘We used to do many things together, such as watching television programmes that we all identified with; we also had games and we would cook Sunday “kos” after church.’ (P8, male, 51 years old)

‘[…*M*]y husband isolates himself and does not want to engage in activities that we used to do as a family.’ (P5, female, 59 years old)

### Relationships with children post-stroke

A total of 50% participants in this study were married to stroke survivors and had children with them. Participants alluded to the tension they observed between their children’s and partners’ relationship post-stroke:

‘His interaction with our children was reduced after the stroke. He became hard on them and they did not understand him. He got along with them more than I did before he had a stroke.’ (P5, female, 59 years old)

Furthermore, participants observed that parents become harsh to their children when they are not understood, straining the child–parent relationship. In addition, participants shared that in some instances stroke survivors felt inadequate as parents as they could no longer fulfil their parental responsibilities and roles as they did prior to the stroke incident. One participant reported the following:

‘She spent more time with children than me because I had two jobs. She was very active, and they played indoor games, such as cards, and now that she has stroke, she is no longer interacting with them the same way. She used to also monitor their school performances. As a result, our lastborn failed because Grade 12 because I cannot do everything in this house.’ (P8, male, 51 years old)

### Influence of stroke on marital or partner relationships

Spousal participants indicated that their relationships with their spouses changed post-stroke, reporting a decrease in intimacy and quality time spent together as married couples. As a result of the stroke impairments, some spouses expressed that their sexual lives completely changed post-stroke as their partners could not satisfy their sexual needs:

‘There is no intimacy or quality time that we spend as a married people… all I do is to take care of her.’ (P8, male, 51 years old)

‘Oh well, you know a man has needs. And those needs can only be fulfilled by their wives. So in my wife’s situation, I cannot expect her to meet my sexual needs.’ (P8, male, 51 years old)

In addition, some female spouses mentioned that their male spouses developed lack of self-confidence as a result of not being able to fulfil some roles. The latter had a negative influence of the marital relationships:

‘I also think he sees himself as useless because he used to work but now he is unable to do so. And that affects his emotions because he is always sad.’ (P5, female, 59 years old)

### Extended family members

Some participants mentioned that family members not residing with the stroke survivor do not visit as frequently as they used to. Therefore, the influence of stroke on family relationships differs. Findings indicated that some family members distanced themselves from the stroke survivor, while others were more supportive and concerned about the stroke survivor and how the immediate family members were coping. However, the reasons for participants’ observations were unclear:

‘His relationship with his family has changed a lot. Some are not visiting us; some would seldom call to check his condition. His parents passed away and I rely on my mother and my daughter.’ (P6, female, 44 years old)

Moreover, another factor that affected relationships was the misconception of the cause of stroke. A spouse of a stroke survivor revealed that she was called names and blamed for the occurrence of the stroke by extended family members. This confirms that some family members do not perceive the stroke as a medical condition. Such perceptions display lack of knowledge about the occurrence of stroke:

‘[… *R*]egarding his family, his older sister used to blame me for my husband’s condition. She would come to our house to utter profanities saying that I am the reason for the stroke.’ (P6, female, 44 years old)

However, some participants observed that their family’s relationships and attitudes towards the stroke survivor remained the same as it was before the stroke. Some participants mentioned that their families developed a supporting net and supported the family member who experienced a stroke:

‘As a family, we supported him and made him feel he is still part of us despite his condition.’ (P2, female, 52 years old)

‘Now my husband has received so much support and my siblings are treating him the same way that they treated him prior to the stroke….’ (P6, female, 44 years old)

Some participants expressed that the stroke incident brought them closer to their partners as they spent more time together than prior to stroke. In this regard, they shared the following experiences:

‘I became close to my wife and very much attentive to the things that must be done as I did before stroke.’ (P4, male, 48 years old)

‘[…*I*]t draws us closer together and now we are spending a lot of time together. I used to visit my friends over weekends and drink. But now I know I must be here to keep her company.’ (P7, male, 37 years old)

### Emotional engagement in caring for a family member with a stroke

Preparing and adjusting the family environment are among other physical measures that may be taken by families to suit the condition of the stroke survivor. This study found that emotional engagement is another aspect that is influenced by stroke. Participants reiterated how emotionally exhausting it is for them care for their family member who experienced a stroke:

‘It gets really difficult at times and I tend to feel very emotionally drained….’ (P5, female, 59 years old)

‘It gets very painful sometimes. I thought that as a parent I would be resting now and that he would be the one taking care of me, not the other way around.’ (P2, female, 52 years old)

Moreover, all the participants expressed that caring for the person with a stroke resulted in psychological trauma because of the constant fear of the unknown. Their fears were mostly related to the sudden or unexpected loss of their family member to stroke. In this regard, a participant shared the following sentiment:

‘I have my days where I worry about what will happen if she were to pass on. I try to be strong for her but sometimes I cannot hold it to myself.’ (P4, male, 48 years old)

### Financial implications of stroke on family functioning

The majority of participants echoed their inability to maintain financial stability post-stroke. Some participants expressed that they had to resign or quit their jobs as there was no one else to care for the stroke survivor:

‘I used to work but now I have to stop and to take care of him. We also have a small business where I sell sweets, chips and small things but it is difficult to run that because when he is not feeling well I must close and take him to the clinic or get an ambulance to take him to the hospital.’ (P3, female, 52 years old)

Small families, especially those consisting of both parents and children, experienced financial setbacks and difficulties because of enforced caregiving responsibilities:

‘I am the only person working here. I have to pay for everything, and we need to have money that is available because sometimes she gets sick and I must be able to pay for transport costs because ambulances take time when we phone them. I have to spend more money on fresh vegetables because she likes them. My son accepted that he cannot have everything he wants because his mother is not well.’ (P7, male, 37 years old)

Participants also shared that during emergencies they had to hire transport as government ambulances would sometimes be delayed in arriving. This compounded their financial burdens. Accordingly, they also struggled to afford a balanced diet for the family member with a stroke, which is essential for a successful recovery:

‘My family does not afford the correct food for her because I am the only one who has a job and I have to pay for other expenses. My son just finished school and I do not have money to pay him to continue his studies. That is how bad our finances are….’ (P4, male, 48 years old)

However, some families established income-generating strategies that included running small businesses, which they found challenging to operate because of their circumstances:

‘[…*H*]e had a job before the stroke. After his stroke, we lost income, and I had to start a business to support the family. However, I cannot run it well because I must make time to care for him. It gets tiresome to balance these demanding tasks.’ (P5, female, 59 years old)

Factors that resulted in financial difficulties included time devoted to caregiving demands and exhaustion and burnout thereafter.

## Discussion

The discussion of this article is presented in context of the McMaster’s modules of family functioning. McMaster’s modules propose six dimensions: problem-solving, communication, roles, affective responsiveness, affective involvement and behaviour control as described by Miller et al. (2000) that can be used to study the family functioning. The findings of this study established that stroke not only affects the life of the stroke survivor but also affects the lives of family members, thus influencing the overall functioning of the family. Caring for a person who has experienced a stroke is a demanding task. This was confirmed by Kumar et al. (2016) who found that life within the family changes post-stroke as family members reorganise their lives to accommodate the stroke survivor. These findings coincide with studies by Guo and Liu (2015), Gillespie and Campbell (2011) and Lutz et al. (2011) who in their research confirmed the significant impact of stroke on the normal functioning patterns of families.

Based on McMaster’s model of family functioning, *communication* is the second dimension that strengthens family functioning. This study found that communication between the stroke survivor and family members was reduced because of the communication barriers. Wray and Clarke (2017:11) explained that communication within the family sometimes became a ‘source of emotional distress, triggering feelings of grief, loss and sadness’. Correspondingly, participants reported that stroke survivors frequently disassociated themselves from engaging with family members because of the communication barriers. Speech impairments following a stroke means that stroke survivors generally struggle to communicate fluently (Vogel, Maruff & Morgan 2010), exacerbating their frustrations even further as they tend to feel judged and inadequate (Winstein et al. 2016). As a result of the communication difficulties, stroke survivors often find themselves protecting their self-identities that are mostly questioned when they struggle to communicate clearly (Wray & Clarke 2017). Participants mentioned that the communication barriers experienced by stroke survivors caused frustration and anger, especially when they were not understood. A chief concern amongst the participants was the reduced interactions. Thus, maintaining flawless communication within families was a challenge as stroke survivors were reported to be easily agitated towards other family members. This finding is supported by Wray, Clarke and Forster (2019) who affirmed that stroke has a significant impact on the family’s communication patterns.

Child–parent relationship is another aspect associated with *roles* – the third dimension of family functioning that was found to be affected by stroke within the family. Participants reported a deterioration in the relationships between stroke survivors and their children post-stroke. This finding is in accordance with Harris and Prvu Bettger (2018) who also found that stroke has a negative impact on the child–parent relationship, respectively. This research reveals that parents struggle to maintain healthy relationships with their children post-stroke. This is because of physical limitations and other stroke-related impairments. Furthermore, participants reported that parental roles and responsibilities are heavily jeopardised by the occurrence of stroke in many ways. As such, the lack of support and parental interaction is reported to have psychological effects that result in poor school performance and social interactions. Kitzmüller et al. (2012) revealed that children with parents who suffered a stroke have concentration problems in schools and this affects their educational outcomes.

In addition, corresponding with a study by Bucki, Spitz and Baumann (2019), this study found that couples often experience changes in their relationships post-stroke because of rapid changes associated with caregiving and nurturing roles. The latter is associated with the first, fourth and fifth dimensions – *problem-solving, affective responsiveness* and *affective involvement*, respectively. According to McMaster’s model, problem-solving and affective responsiveness is based on the family’s ability to address present challenges in a constructive manner (Miller et al. 2000). Participants reported that they experienced emotional disconnection with their partners as communication was not as flawless as it was pre-stroke. Communication barriers after stroke were identified as key factors limiting stroke survivors’ engagement with their partners. This finding is in accordance with Kitzmüller et al. (2012:8) who also found that the ‘lack of communication because of aphasia caused emotional problems, misunderstandings and feelings of loneliness within the couples’. Furthermore, participants also reported that their partners who experienced a stroke tended to isolate and exclude themselves from family activities. Consequently, their self-isolation resulted in emotional disconnection and a decrease in family quality time. Kitzmüller et al. (2012) found that stroke affects spousal relationships negatively as one spouse usually assumes multiple caregiving roles, resulting in the overburden of responsibilities. A further finding of this study was that intimacy between partners may be disrupted as one spouse may be struggling to function optimally. This finding is supported by Gillespie and Campbell (2011) who found that spousal relationships may be faced with challenges, especially when it comes to intimacy.

This study also found that because of the sudden occurrence of stroke, some family members developed misconceptions about the cause of the stroke resulting in conflict. This highlighted another aspect of family functioning associated with *behaviour control* – the sixth dimension of family functioning – interprets the interpersonal behaviour within the family system. According to Miller et al. (2000), some family members may develop misunderstandings during the process of re-defining the family’s functional patterns. In such cases, spouses were often blamed for the stroke of their loved one. Accordingly, Wegner and Rhoda (2015) reported that factors such as religious beliefs, social norms and traditions contribute to the perceptions and understandings of diseases, including stroke. This, therefore, does not only show misconceptions of stroke but also reveals a lack of education about diseases in some contexts.

It was further established that the burden of stroke does not only affect children and spouses but the whole family system is impacted. The research promulgates that relationships within the family system may be affected in one of two ways. On the one hand, the caregiving responsibilities may place a lot of strain on family relationships as family members put their lives on hold to assume new caregiving responsibilities. In accordance with McMaster’s model, this is linked with *roles* – the third dimension of family functioning – which ensures the fulfilment of essential roles for the day-to-day activities of the family system (Miller et al. 2000). This finding is corroborated by Mishra, Mishra and Gajjar (2016) who reported that families of stroke survivors may experience greater caregiving burden because of their altered roles and responsibilities, especially when they are not well organised. This in turn may give rise to family dysfunction (Kitzmüller et al. 2012). On the other hand, some participants reported a positive experience in relation to the burden of stroke. They stipulated that their families spent more time together than they did prior to the stroke. Others remarked that they integrated the stroke survivor in their family processes and thereby refrained from discriminating against them because of their health conditions. These findings resonate with that of Simeone et al. (2015) that in some instances some families developed greater affection through the support system offered to the stroke survivor, which in turn strengthened family relationships. This is confirmed by Mohammadi et al. (2017) who stated that functional families foster healthy environments for stroke survivors to communicate freely about problems and fears and thereby provide better support to their families.

These authors assert that the latter creates a positive environment that facilitates the recovery process (Mohammadi et al. 2017). In the light of family systems theory, some families have high responsiveness towards helping and empowering members during adversities. Despite the family’s ability to establish a stroke support system during adversities, this study along with that of Torregosa et al. (2018) found that the long-term trajectory of stroke may significantly change functional patterns within the family and compromise its ability to care for other vulnerable members. This study and Gillespie and Campbell (2011) and Owolabi et al. (2015) found amongst other factors that mostly impair family functioning include the cost of caring for the stroke survivors. Gillespie and Campbell (2011) in their study further reveal that caring for a person who experienced a stroke requires financial means and as such families in low socioeconomic statuses may experience financial strains. A study by Rhoda (2014) confirmed that the general population residing in the peri-urban parts of the Western Cape, South Africa experience financial strains because of unemployment amongst other factors. In this study, two of the eight participants were employed on contractual bases, while the remaining six were unemployed.

### Limitations of the study

Findings discussed in this study described the experiences of families of stroke survivors from a low socio-economic background. Such experiences may differ in other contexts or with other individuals from backgrounds with a different socio-economic status. In addition, having a diverse study sample might have represented a varied demographic South African profile. In addition, the sample size of this study may also be a limitation. Therefore, the influence of stroke on family functioning can be contextually interpreted.

## Conclusion

The focus of this article was on family functioning after a family member has experienced a stroke. The perspectives of family members revealed that stroke becomes a burden to the family and has a significant impact on their daily functioning. To deal with their new reality, families had to implement changes and adopt new functional approaches to accommodate their family member who experienced a stroke. It was established that stroke negatively impacts six dimensions of family functioning. These are: (1) problem-solving, (2) communication, (3) roles, (4) affective responsiveness, (5) affective involvement and (6) behaviour control. Despite negative reports, some participants indicated spending more time together as a family post-stroke and explored ways of integrating the stroke survivor in their family processes. Misunderstandings regarding stroke also surfaced, creating disorder within some families as immediate and extended family members tried to find closure. Another aspect that affects the functioning of stroke survivors and their families includes limited financial and essential resources not only for the stroke survivors but also for other family members that are affected. This highlights the importance of research to not only examine the impact of stroke on family members who experienced a stroke and those who assume caregiving roles but to also consider the perceptions and experiences of immediate and extended family members as well. This will shed much needed light on the neglected research area of the nature and influence of stroke on family members.

### Recommendations

Through this study, it became clear that there is a dearth of studies that have specifically assessed the areas of family functioning impacted by stroke. Recommendations derived from the findings of this study include financial relief of stroke survivors and their families. It was also evident that families of stroke survivors have limited knowledge about stroke. This is an area that requires attention as it can assist families of stroke survivors to understand stroke better. Lastly, research that considers the influence of stroke on the broader family is needed to plan appropriate interventions to assist families of stroke survivors to establish and maintain a conducive family environment during the process of caring for the stroke survivors.

## Appendix 1: Interviewing guide

### The survivor’s limitations after stroke

- Can you please tell me about the activities that the survivor used to do pre-stroke?
- What was the initial role of the survivor within the family?
- What are other limitations that your partner is experiencing as a result of stroke?
- Do you think your partner would ever be able to fulfil some of the roles he or she used to assume?
- How has that impacted on your family functioning?
- Are there any adjustments that the family has made as to adjust to stroke within the family?

### The stroke survivor’s health-related quality of life after stroke

- Are there health-related challenges that have been brought about by stroke in the survivor’s life?
- How would you describe the health status of the stroke survivor after the stroke?
- How is the survivor affected by stroke?
- If there are physical and mental implications of stroke for the survivor’s life, what do you do then as a caregiver to ensure a stable sense of well-being for the survivor?
- As part of ensuring the survivor’s good health state, what would you say works and does not work?
- Would you say that the identity of the survivor has been impacted by the incident of stroke?
- Would you say that the survivor’s self-concept is affected by stroke?

### Survivor’s interfamily relationships (with children if there are any, parents, siblings and extended family members) functioning

- Let us talk about the impact of stroke on family relationships, tell me about the relationships the survivor has and had before stroke with family members
- Do you think that stroke has impacted on relationships within the family?
- As a family have you managed to preserve the nature of relationships within your family?
- What are your family members doing to ensure that the stroke survivor is integrated within the family processes?
- Tell me about the attitudes of your family members towards the survivor as a person living with stroke
- Do you notice any change in the relationships that the survivor has with other family members?
- Besides the people living with the stroke survivor, how involved are other family members in the life of the survivor?
- Is there something that you would like to share that I did not ask on the impact of stroke on familial relationships?

#### The impact of stroke on marital relationships (*Applicable to married or cohabitating participants*)

- Can you please reflect on the nature of the relationship you had with the stroke survivor prior to the stroke event?
- Has that relationship changed in some way because of the event of stroke?
- Do you think that your marital relationship is still healthy?
- Can you briefly comment on the resilience that your marital relationship has displayed during after stroke?
- Are there aspects of your relationship that has been affected by the incident of stroke?

### The impact of stroke on family members

- Stroke is a traumatic medical incident. Are there some things in particular that you and your family members do to deal with it?
- Who would you say is mostly implicated by stroke in your family?
- How do you think the lives of those implicated by stroke have changed?
- Are there things that the individuals or you are doing to ensure that they cope with stroke?

### The survivor’s post stroke social activities

- Were there social activities that the survivor was involved in before stroke?
- Is the survivor still active or involved in any of these social activities?
- If not, do you think that impacts or influences how the stroke survivor views themselves in any form?
- Would you now say after 6 months the survivor has recovered and is showing interest on being involved socially?
- What is the attitude of community members towards the survivor?
- Finally, do you think being active or involved in social activities would or has a positive impact on the recovering process of the survivor?

### Family functioning during stroke

- Family functioning refers to how the family operates and support each other. How would you say your family functioning is influenced by stroke?
- What adjustments did the family have to make to cope with the experiences of having to live with a person that had a stroke?
- Are the family finances the only aspect that is influenced by stroke?
- Were there activities that you used to do as a family? If so, are those still done now that there is an individual that has a stroke?
- Let us talk about decision making processes. How does the family manage such processes now that there has been stroke?
- I know that everyone experiences stroke differently. How would you describe the experience of other family members?
- What areas of functionality in your family are most influenced or impacted by stroke?
- What has your family done to deal with some of the unexpected changes that may have been brought about by the event of stroke?

## Appendix 2

**TABLE 1-A2:** Demographic information of the participants

Participant number	Gender	Caregiver’s age	Survivor’s age	Education level of carer	Employment status	Relationship to survivor	Size of family
1	Female	42	84	Diploma	Employed	Child	4
2	Female	52	33	Grade 7	Unemployed	Mother	12
3	Female	52	49	Grade 12	Unemployed	Sister	4
4	Male	48	42	Grade 12	Employed	Spouse	3
5	Female	59	60	Grade 12	Unemployed	Spouse	5
6	Female	44	52	Grade 12	Unemployed	Spouse	3
7	Male	37	43	Grade 10	Unemployed	Spouse	3
8	Male	51	46	Grade 8	Unemployed	Spouse	7

*Source:* This table was developed by authors for the purpose of collecting and organising information of the participant.
